# Study of intra-abdominal hypertension prevalence and awareness level among experienced ICU medical staff

**DOI:** 10.1186/s40779-016-0097-y

**Published:** 2016-09-12

**Authors:** Hua-yu Zhang, Dong Liu, Hao Tang, Shi-jin Sun, Shan-mu Ai, Wen-qun Yang, Dong-po Jiang, Lian-yang Zhang

**Affiliations:** Trauma Center, State Key Laboratory of Trauma, Burns and Combined Injury, Institute of Surgery Research, Daping Hospital, Third Military Medical University, Chongqing, 400042 China

**Keywords:** Intra-abdominal pressure, Intravesical pressure, Intra-abdominal hypertension, Abdominal compartment syndrome, Questionnaire

## Abstract

**Background:**

Intra-abdominal hypertension (IAH) is a disease with high morbidity and mortality among critically ill patients. The study’s objectives were to explore the prevalence of IAH and physicians’ awareness of the 2013 World Society of Abdominal Compartment Syndrome (WSACS) guidelines in Chinese intensive care units (ICUs).

**Methods:**

A cross-sectional study of four ICUs in Southwestern China was conducted from June 17 to August 2, 2014. Adult patients admitted to the ICU for more than 24 h, with bladder catheter but without obvious intravesical pressure (IVP) measurement contraindications, were recruited. Intensivists with more than 5 years of ICU working experience were also recruited. Epidemiological information, potential IAH risk factors, IVP measurements and questionnaire results were recorded.

**Results:**

Forty-one patients were selected. Fifteen (36.59 %) had IVP ≥ 12 mmHg. SOFA (Sequential Organ Failure Assessment) hepatic and neurological sub-scores were utilized as independent predictors for IAH *via* logistic backward analysis. Thirty-seven intensivists participated in the survey (response rate: 80.43 %). The average score of each center was less than 35 points. All physicians believed the IAH prevalence in their departments was no more than 20.00 %. A significant negative correlation was observed between the intensivists’ awareness of the 2013 WSACS guidelines and the IAH prevalence in each center (*r* = -0.975, *P* = 0.025).

**Conclusions:**

The prevalence and independent predictors of IAH among the surveyed population are similar to the reports in the literature. Intensivists generally have a low awareness of the 2013 WSACS guidelines. A systematic guideline training program is vital for improving the efficiency of the diagnosis and treatment of IAH.

## Background

Scholars have long studied the effects of intra-abdominal pressure (IAP) on human physiological functions [1]. In recent years, studies have increasingly discussed intra-abdominal hypertension (IAH) and abdominal compartment syndrome (ACS) [2]. Numerous clinical research studies have shown the harmful effects of increased IAP on multi-system functions and organ systems such as the respiratory, circulatory and renal systems [3]. Epidemiological studies have found a high prevalence of IAH among critically ill intensive care unit (ICU) patients [4] and have shown that the occurrence of IAH is an independent predictor of mortality [5]. The studies note that after IAH progresses to ACS, a patient’s mortality can be as high as 80.00 % [5].

Before the publication of the first-edition guideline by the WSACS in 2006 [6], there was no consensus among scholars regarding the diagnostic criteria for IAH and ACS, the standard IAP measurement protocol, or the treatment strategy. Ravishankar et al. [7] surveyed British physicians to determine their awareness of IAH and its treatment and reported an urgent need for guidelines. After publication of the 2006 guidelines, intravesical pressure (IVP) measurement was established as the gold standard for IAP monitoring. However, there was still room for improvement in the diagnostic threshold for IAH, IVP measurement indications, and for the timing of surgical intervention [8]. The 2013 WSACS guidelines follow the former definitions of IAH and ACS, extend new concepts such as abdominal compliance, and evaluate the treatment strategy based on the GRADE (Grading of Recommendations, Assessment, Development, and Evaluation) system [9]. Although ICU physicians attach importance to IAH, their implementation of the guidelines is not satisfactory [10].

Through this survey, we sought to explore the prevalence of IAH among critically ill patients in China and to investigate whether any progress has been achieved in the awareness of IAH-related knowledge since the survey conducted by Zhou et al. [8] in 2010.

## Methods

### Epidemiological investigation method

Based on the 2013 WSACS guidelines, a series of 24-h cross-sectional studies were conducted from June 17 to August 2, 2014 in four trauma/emergency/general ICUs of Southwestern China (three teaching hospitals and one regional emergency medical center).

### Patients and data collection

Participant inclusion criteria included age greater than 18 years, admission to ICU (elective/emergency laparotomy or internal diseases such as acute severe pancreatitis, hepatic failure and renal dysfunction, etc.) for more than 24 h prior to the survey, presence of urinary bladder catheter, and no obvious measurement contraindications during the survey period. Patients or family members who did not agree to participate in this survey were excluded. Survey data included demographic information (gender, age, height, weight, length of ICU stay prior to the survey, cause of ICU admission), physical examination and laboratory data, and the patient’s potential risk factors according to the 2013 WSACS guidelines. Patients underwent standard IVP measurement, which was performed by investigators. The manometry device modified by Malbrain et al. [11] was used and assembled and connected to the urinary catheter under sterile conditions. Patients were placed in a complete supine position, and 20 ml of sterile saline was injected into the bladder *via* the catheter after emptying. The midaxillary line was set as the zero reference plane, and the IVP value was read at the end of the measurement period by central venous pressure monitoring sets (Medifix, B.Braun Melsungen AG, Melsungen, Germany), expressed in mmHg (1 mmHg = 1.36 cmH_2_O). The frequency of IVP measurements was once every 4 h. Each measurement was repeated within a three-minute interval, and the average was used as the measurement value to minimize reading errors.

### Questionnaire survey

The epidemiological investigation and questionnaire study of each ICU was performed in the same survey period. The paper-and-pencil questionnaires were completed by intensivists with ICU working experience of more than 5 years to determine their awareness of the 2013 WSACS guidelines. The questionnaire had a total score of 100 points and was divided into three types of questions: 15 points for true or false, 45 for single-choice, and 40 for multiple-choice. Topics included basic IAH/ACS concepts, methods and indications of IAP monitoring, and non-surgical and surgical treatment strategies. Intensivists were also asked to speculate about the IAH prevalence in their departments.

### Statistical analysis

Measurement data were expressed with the mean ± SD or median (interquartile range). Continuous variables with normal distribution were compared using a* t* test. Abnormally distributed variables were compared using the Mann-Whitney *U* test. Multiple groups were compared using One-way ANOVA and the Kruskal-Wallis *H * test. Frequencies were compared using the Pearson Chi-Square test and Fisher’s exact test. Logistic backward regression was used to analyze the independent risk factors of IAH. A linear correlation and regression analysis was applied to define the relationship between questionnaire scores and IAH prevalence. *P* <0.05 was considered statistically significant. The software SPSS 13.0 (SPSS, Chicago, IL) was used for statistical analysis.

## Results

Among the 64 patients in the four ICUs, 41 met the inclusion criteria (64.06 %) and 23 (35.94 %) without bladder catheters were excluded. No significant difference in demographic information between the IAH and non-IAH groups was observed (Table 1). The average IAH prevalence among surveyed patients was 36.59 %, but no significant difference in IAH prevalence among the four surveyed centers was noted (*P* = 0.447, Table 2).

**Table 1:** Demographic information of surveyed patients

Item	Total (*n* = 41)	IAH (*n* = 15)	Non-IAH (*n* = 26)	*P* value
IAP (mmHg)	10.00 ± 4.35	14.40 ± 3.07	7.46 ± 2.58	0.000
Age (year)	57.17 ± 16.28	53.40 ± 13.69	59.35 ± 17.48	0.265
Male [*n* (%)]	27 (65.85)	10 (66.67)	17 (65.38)	0.934
BMI (kg/m^2^)	22.44 ± 3.26	22.51 ± 2.70	22.40 ± 3.59	0.916
Pre-study ICU stay (d)	7.00 (2.00–20.50)	6.33 ± 5.15	10.50 (2.00–55.75)	0.089
Admission reason (medicine/surgery)	22/19	6/9	16/10	0.183

*BMI* body mass index

**Table 2:** IAH prevalence of the 4 surveyed ICUs

Unit	Total (*n*)	IAH [*n* (%)]
ICU A	14	5 (35.71)
ICU B	8	2 (25.00)
ICU C	6	4 (66.67)
ICU D	13	4 (30.77)
Total	41	15 (36.59)

The 2013 WSACS guidelines divide the IAH risk factors into five categories with 34 sub-items, including decreased abdominal wall compliance, increased gastrointestinal contents, increased abdominal contents, capillary leak/fluid resuscitation, and other/miscellaneous. Univariate analysis indicated a significant difference in abdominal expansion, SOFA score and hepatic/cardiovascular/neurological sub-scores between the IAH and non-IAH groups (Table 3). The logistic backward regression analysis showed that the SOFA hepatic sub-score (OR= 18.281, 95 % CI: 1.645-203.207, *P* = 0.018) and the neurological sub-score (OR= 7.317, 95 % CI: 1.569-34.126, *P* = 0.011) were independent risk factors for IAH.

**Table 3:** Disease severity scores of surveyed patients

Item	Total (*n* = 41)	IAH (*n* = 15)	Non-IAH (*n* = 26)	*P* value
APACHEII score	13.95 ± 4.97	15.53 ± 5.15	13.04 ± 4.73	0.123
SOFA score	4.66 ± 3.75	7.47 ± 3.70	3.04 ± 2.71	0.000
Respiratory	0.00 ± 0.00	0.00 ± 0.00	0.00 ± 0.00	1.000
Cardiovascular	0.00 (0.00–1.00)	1.80 ± 1.82	0.00 (0.00–0.00)	0.002
Renal	0.00 (0.00–1.00)	0.00 (0.00–1.00)	0.00 (0.00–0.25)	0.715
Coagulation	0.00 (0.00–1.00)	1.07 ± 1.28	0.00 (0.00–1.00)	0.260
Hepatic	0.00 (0.00–1.00)	1.40 ± 1.12	0.00 (0.00–1.00)	0.001
Neurological	2.00 (0.00–3.00)	3.00 (3.00–3.00)	1.19 ± 1.23	0.000

Forty-six ICU staff met the inclusion criteria and participated in the survey, of whom 37 (80.43 %) completed and submitted the questionnaire. There was no significant difference in demographic information or average questionnaire scores (*P* = 0.976) among the four centers (Table 4).

**Table 4:** Demographic information of ICU medical staff and questionnaire score

Item	ICU A (*n* = 19)	ICU B (*n* = 7)	ICU C (*n* = 4)	ICU D (*n* = 7)
Male [*n* (%)]	10 (52.63)	3 (42.86)	2 (50.00)	0 (0.00)
Age (year)	33.47 + 7.00	41.14 + 10.71	36.25 + 7.93	38.00 + 5.10
Doctors/nurses	14/5	4/3	2/2	5/2
ICU working time (year)	9.16 + 4.76	15.07 + 10.05	11.13 + 7.53	7.57 + 2.94
Average score	30.68 ± 14.72	33.00 ± 11.73	27.75 ± 26.35	31.43 ± 15.09

**Fig. 1:**
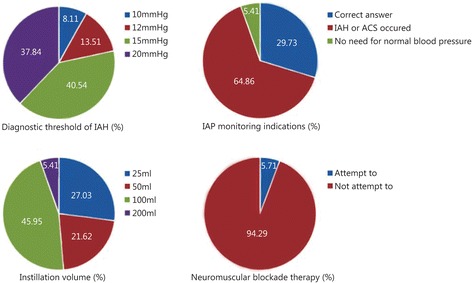
Details of some main questions

Questionnaire contents were categorized by basic IAH concepts, standard IAP monitoring procedures and treatment protocol. The “correct” rate of each question was relatively low (Table 5). Figure 1 describes some of the main questions in detail.

**Table 5:** Questionnaire on awareness of WSACS 2013 guidelines

Item	Number of correct answer	Percentage (%)
IAH basic concepts (5 questions)		
IAP fluctuation range of severe patients	13	35.14
Primary ACS	16	43.24
Diagnostic threshold value of IAH	5	13.51
Normal abdominal perfusion pressure	21	56.76
Risk factors	9	24.32
IAP monitoring (4 questions)		
Standard IAP measurement	12	32.43
Maximum amount of saline injected into bladder	10	27.03
IAP monitoring indications	11	29.73
IAP monitoring frequency	23	62.16
Non-surgical/surgical treatment (3 questions)		
Non-surgical treatment	2	5.41
Temporary abdominal cavity closure technology	11	29.73
Definitive abdominal wall reconstruction indications	11	29.73

Twenty-seven ICU physicians (72.97 %) estimated the prevalence of IAH in their department to be less than 10 %, of whom 22 (81.48 %) estimated it to be no more than 5 %. The remaining 10 (27.03 %) estimated the prevalence to be within 10 % to 20 %, and none estimated it to be higher than 20 %.

The linear correlation and regression analysis of the average questionnaire score of intensivists and the IAH prevalence of each ICU showed a significantly negative correlation between the awareness level of the 2013 WSACS guidelines and IAH prevalence (*r* = -0.975, *P* = 0.025). The regression equation was *Y* (IAH prevalence) = 292.874-8.248 *X *(awareness level) (R^2^ = 0.951, *F *= 38.813, *P* = 0.025).

## Discussion

The study of epidemiology is crucial for identifying the distribution and determinants of disease in a specific population. WSACS attaches great importance to epidemiological studies among various types of patients. Recent epidemiological data about critically ill patients have come mainly from western countries. The prevalence rates of IAH vary significantly in the literature due to different statistical strategies and IAP measurement methods. IAH prevalence fluctuates from 17.5–63.86 % [5, 12] but is mainly concentrated at approximately 30 % [13, 14]. The IAH prevalence (36.59 %) of the present study is similar to that reported in the literature, which reflects a high risk among people of different countries and races. We found that patients with IAH had higher SOFA scores than non-IAH patients, which is consistent with the literature [5]. However, there is still a lack of epidemiological data about critically ill Chinese IAH patients.

Awareness of IAH risk factors is the foundation of disease prevention. As early as 2006, WSACS recognized the importance of IAH with the slogan, “It’s time to pay attention!” [15]. The list of risk factors published in the 2013 WSACS guidelines was established on the basis of a series of studies [16]. We screened the specific risk factors of the surveyed patients. Although we did not enlarge the spectrum of IAH risk factors, our results may help to clarify the epidemiological features of certain populations and to enrich the database of distribution of the risk factors among different races and countries. Our finding is similar to that reported by Blaser et al. [14], namely, that the SOFA liver sub-score is an independent predictor of IAH. In addition, we found that the SOFA neurological sub-score is also an independent IAH risk factor. A possible explanation may be that some unconscious patients experience hyperventilation, and the resulting tightness of the abdominal muscle may increase the IAP. As additional studies are performed, we believe that more potential risk factors will be revealed and that the strategy of disease prevention will become more comprehensive.

Analysis of the results of the questionnaires indicates that the awareness of the 2013 WSACS guidelines among Chinese ICU physicians is in urgent need of improvement. Because a sound understanding of IAH-related risk factors and IVP measuring indicators is crucial to avoid missed diagnosis, misdiagnosis, and iatrogenic injuries [17], the guidelines recommend measuring IVP when any known risk factor is present [9]. Unfortunately, only 24.32 % of respondents had a clear awareness of the risk factors. For example, 64.86 % chose to measure IVP only in highly suspected IAH patients. Although the result is better than in a previous study [8], it does not conform to the requirements of the guidelines and the principle of “early detection, early diagnosis and early treatment.” Our finding suggests that ignorance of the potential IAH risk factors (increased head of bed angle, massive fluid resuscitation, etc.) may lead to disease progression. It also means that the intervention time is delayed, the burden on both patients and intensivists is increased, and the outcomes are adversely affected.

It has long been known that IVP monitoring is essential to IAH prevention and timely treatment. The literature has confirmed that pathologically elevated IAP can cause various adverse effects on physical functioning [18]. The presence of IAH in the first 24 h after ICU admission is also an independent predictor of mortality [5]. A standard IVP measurement method is the most reliable way to diagnose IAH and ACS, and thorough mastering of diagnostic criteria is the key to making a correct diagnosis and choosing an appropriate treatment strategy. Research has shown that an injection volume exceeding 25 ml may overestimate the actual reading of IVP [19]. Nevertheless, 27 physicians (72.97 %) chose an excessive amount of injection fluid. A significant majority of intensivists (86.49 %) selected the incorrect pressure threshold for diagnosing IAH, which, more than anything else, accounts for missed diagnosis and misdiagnosis. IVP measurement has also been accepted as the gold standard of IAP monitoring due to its simplicity of operation, accurate results, low cost and high repeatability [16].

Scholars believe that greater understanding of IAH among intensivists, standard and reasonable IVP monitoring of high-risk patients, and timely intervention with a goal-directed resuscitation strategy may improve short- and long-term outcomes [20, 21]. However, most of the physicians in our study (72.97 %) optimistically estimated the IAH prevalence in their ICU to be no more than 10.00 %, and none thought it would exceed 20.00 %. The high prevalence of IAH in a single center and the total prevalence of IAH (66.67 % and 36.59 %, respectively) both indicate that China is not a paradise free of IAH. These findings suggest that insufficient knowledge will lead to minimizing the severity of the disease.

Although most of the treatment strategies recommended by the 2013 WSACS guidelines graded low levels of evidence as relevant [9], every therapeutic option is supported by clinical or laboratory studies. Either neglecting or inappropriately applying certain interventions can delay treatment, resulting in additional damage to the patients. A certain degree of progress has been achieved by Chinese intensivists since the 2010 study [8], especially in the indicator selection and performance of IVP measurement. However, timely monitoring, standardized IVP measurement and compliance with diagnostic criteria are still the core problems in IAH prevention and treatment.

Compared with the severity of IAH and the difficulty of treatment, practitioners’ understanding of IAH is still at a relatively low level. The main reason is the lack of effective guidelines training plans. Better understanding can be promoted by the following methods.

1. Proceedings of case reports. Each case report can be organized in the following sections: clinical presentation, examinations (physical/laboratory/radiological/IAP monitoring), diagnosis and etiology, and treatment and prognosis. Expert comments on the timing and method of each section should be available. This is the most expedient way to become familiar with the features and intervention strategy of IAH. In addition, commentaries are helpful in recognizing the key points and potential drawbacks of the IAH management algorithm.
2. Continuing education. The continuous renewal of knowledge is the key to enhancing professional proficiency. Regular training in IAH-related knowledge should be weighed as an important factor in the certification and qualification of intensivists. Each ICU should be invited to make suggestions in its annual training plan and to host training courses.
3. Academic journals. There are still many unanswered questions regarding the scope of pathogenesis and treatment strategy. For example, there has been increasing attention to the issue of abdominal compliance because it has been one of the most neglected parameters. As the authority for IAH research, the WSACS is capable of publishing an academic journal focusing on IAH and relevant diseases. With the increasing number of articles being published, more attention should be paid to this area and encouragement for research should be given. Greater depth and breadth in IAH research will provide more sufficient data for updating prevention and intervention strategies.

## Conclusions

Through an analysis of the relationship between IAH prevalence and its awareness, we demonstrated the important role that guidelines play in the diagnosis and treatment of IAH. The prevalence of IAH in Chinese ICUs (36.59 %) is consistent with that in the literature. Patients with high SOFA liver/neurological sub-scores are prone to developing IAH. Lack of awareness of IAH and insufficient compliance with the 2013 WSACS guidelines are the main reasons for the underestimation of IAH.

There are several limitations to this study. Due to its cross-sectional nature, we could only obtain a snapshot of the clinical information within a certain period. The lack of exact outcomes makes it difficult to specify the relationship between intensivists’ awareness and prognoses such as mortality. Notably, the higher the level of understanding of the WSACS guidelines is, the more effective the intervention, the stronger the vigilance, and the earlier the IVP monitoring and treatment. Chinese intensivists have a similar workload as their counterparts elsewhere, requiring them to continuously improve their IAH-related knowledge. Enhancing awareness of the updated WSACS guidelines is essential to promoting clinical efficiency, promoting the efficiency of disease treatment, and improving patient prognosis. The above suggestions could be of great importance in improving prognosis and reducing morbidity and mortality.

## Abbreviations

ACS: Abdominal compartment syndrome
GRADE: Grading of recommendations, assessment, development and evaluation
IAH: Intra-abdominal hypertension
IAP: Intra-abdominal pressure
ICU: Intensive care units
IVP: Intravesical pressure
SOFA: Sequential organ failure assessment
WSACS: World Society of Abdominal Compartment Syndrome
